# Recombinant Irisin Prevents the Reduction of Osteoblast Differentiation Induced by Stimulated Microgravity through Increasing β-Catenin Expression

**DOI:** 10.3390/ijms21041259

**Published:** 2020-02-13

**Authors:** Zhihao Chen, Yan Zhang, Fan Zhao, Chong Yin, Chaofei Yang, Xue Wang, Zixiang Wu, Shujing Liang, Dijie Li, Xiao Lin, Ye Tian, Lifang Hu, Yu Li, Airong Qian

**Affiliations:** 1 Lab for Bone Metabolism, Key Lab for Space Biosciences and Biotechnology, School of Life Sciences, Northwestern Polytechnical University, Xi’an 710072, China; chzhh@mail.nwpu.edu.cn (Z.C.); zhangyan0911@mail.nwpu.edu.cn (Y.Z.); sofan@mail.nwpu.edu.cn (F.Z.); yinchong42@mail.nwpu.edu.cn (C.Y.); yangchaofei@mail.nwpu.edu.cn (C.Y.); wangxue1005@mail.nwpu.edu.cn (X.W.); wuzx@mail.nwpu.edu.cn (Z.W.); liangsj@mail.nwpu.edu.cn (S.L.); lidijie@mail.nwpu.edu.cn (D.L.); linxiao@nwpu.edu.cn (X.L.); tianye@nwpu.edu.cn (Y.T.);; 2 Xi’an Key Laboratory of Special Medicine and Health Engineering, School of Life Sciences, Northwestern Polytechnical University, Xi’an 710072, China; 3 Research Center for Special Medicine and Health Systems Engineering, School of Life Sciences, Northwestern Polytechnical University, Xi’an 710072, China; 4 NPU-UAB Joint Laboratory for Bone Metabolism, School of Life Sciences, Northwestern Polytechnical University, Xi’an 710072, China

**Keywords:** irisin, simulated microgravity, osteoblast differentiation, β-catenin, bone loss

## Abstract

*Background*: Irisin, a novel exercise-induced myokine, was shown to mediate beneficial effects of exercise in osteoporosis. Microgravity is a major threat to bone homeostasis of astronauts during long-term spaceflight, which results in decreased bone formation. *Methods*: The hind-limb unloading mice model and a random position machine are respectively used to simulate microgravity in vivo and in vitro. *Results*: We demonstrate that not only are bone formation and osteoblast differentiation decreased, but the expression of fibronectin type III domain-containing 5 (Fdnc5; irisin precursor) is also downregulated under simulated microgravity. Moreover, a lower dose of recombinant irisin (r-irisin) (1 nM) promotes osteogenic marker gene (alkaline phosphatase (*Alp*), collagen type 1 alpha-1(*ColIα1*)) expressions, ALP activity, and calcium deposition in primary osteoblasts, with no significant effect on osteoblast proliferation. Furthermore, r-irisin could recover the decrease in osteoblast differentiation induced by simulated microgravity. We also find that r-irisin increases β-catenin expression and partly neutralizes the decrease in β-catenin expression induced by simulated microgravity. In addition, β-catenin overexpression could also in part attenuate osteoblast differentiation reduction induced by simulated microgravity. *Conclusions*: The present study is the first to show that r-irisin positively regulates osteoblast differentiation under simulated microgravity through increasing β-catenin expression, which may reveal a novel mechanism, and it provides a prevention strategy for bone loss and muscle atrophy induced by microgravity.

## 1. Introduction

Disuse osteoporosis is a worldwide clinically relevant problem for those with a lack of mechanical load (e.g., bedridden, microgravity, aging) [1,2,3,4]. It is well known that microgravity results in decreased bone mineral density (BMD) and leads to bone loss during long-term spaceflight [5]. Bone, as a specifically gravity sensory organ, is mainly composed of osteocytes and osteoblasts that respond to and adapt to changes in gravity [6,7]. Many studies demonstrated that simulated microgravity leads to decreased osteoblast differentiation or bone formation [8,9,10], while the cellular and molecular mechanisms are not yet completely understood. 

According to reports, there are many osteoporosis risk factors such as cytokines (e.g., interleukin) [11,12], hormones (e.g., parathyroid hormone) [13], and signaling pathways molecules (e.g., wingless-type MMTV integration site family (Wnt), bone morphogenetic protein(BMP)) [14,15], regulating bone metabolism under microgravity environment. Skeletal muscles and bone, as neighboring tissues, are the two largest tissues in the musculoskeletal system [12]. Muscle atrophy preceding bone loss is also induced by microgravity [16]. Especially for long-duration spaceflight astronauts, recovery from muscle atrophy is faster than that from bone loss [17]. There is increasing evidence that muscle-derived humoral factors, i.e., myokines (a kind of cytokine released from muscle), serve as important regulators of bone metabolism [12,18], such as interleukin-6 (IL-6), osteoglycin, and irisin [19].

Irisin is a novel hormone-like molecule released from skeletal muscle after physical exercise. It is cleaved from the extracellular domain of the transmembrane receptor fibronectin type III domain-containing 5 (FNDC5) and secreted from skeletal muscle into the bloodstream [20]. A positive correlation between irisin level and BMD was reported in the serum of young women [21] and plasma of Chinese elderly men [22]. Recent studies indicated that irisin displays anabolic actions on the skeleton through the stimulation of osteoblast differentiation and bone formation [23,24,25,26,27]. Significantly, Colaianni et al. found that recombinant irisin (r-irisin) could effectively prevent bone loss induced by hind-limb unloading in vivo. The BMD of both the cortical and the trabecular bone volume fraction (BV/TV) are also prevented by r-irisin injection [26]. In addition, FNDC5 messenger RNA (mRNA) level was decreased in the soleus muscle of hind-limb unloading mice. Furthermore, regression analysis suggested that the FNDC5 level of the soleus muscle was positively related to tibia trabecular BMD [28]. Therefore, the mechanism of irisin in osteoblast differentiation and bone formation under microgravity warrants an investigation.

Previous studies showed that Wnt/β-catenin signaling is a key signaling pathway in osteoblast differentiation and bone formation [29,30,31,32,33]. In osteoblasts, mechanical stimulation causes a rapid, transient accumulation of active β-catenin in the cytoplasm and its translocation into the nucleus [34,35], and β-catenin is also sensitive to simulated microgravity [14,36]. It was reported that irisin influences adipogenesis through regulating the Wnt/β-catenin signaling pathway [37], and it increases β-catenin expression during osteogenic differentiation [27]. These findings bring up an intriguing possibility that irisin may be involved in the process of osteoblast differentiation through altering β-catenin under simulated microgravity condition.

In this study, the hind-limb unloading mice model and a random position machine (RPM) were used to simulate microgravity in vivo and in vitro, respectively. Here, we firstly identified that Fdnc5 (irisin precursor) expression was decreased and positively correlated with osteoblast differentiation and bone formation under simulated microgravity environment. Furthermore, the gain of function of irisin in primary osteoblast differentiation under the simulated microgravity environment was confirmed after treatment with r-irisin. Lastly, we demonstrated the mechanism of r-irisin preventing reduction of osteoblast differentiation induced by simulated microgravity through increasing β-catenin expression after treatment with r-irisin. Our finding may reveal a novel mechanism for osteoporosis induced by microgravity, and it provides a potential therapeutic strategy for the pathological musculoskeletal disorders.

## 2. Results

### 2.1. Simulated Microgravity Inhibits Irisin Precursor Expression, Bone Formation, and Osteoblast Differentiation

**Figure 1:**
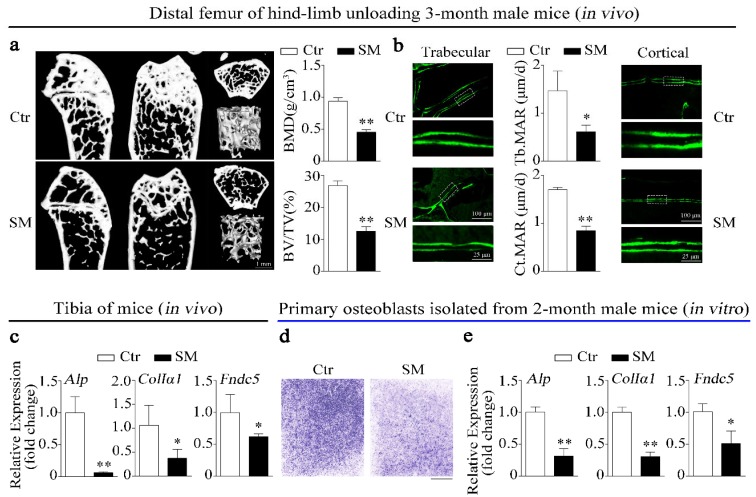
Irisin level, bone formation, and osteoblast differentiation were decreased under simulated microgravity in vivo and in vitro. (**a**) Representative images showing three-dimensional trabecular architecture in distal femur and microCT analysis of microstructural bone parameters of the distal femurs including BMD (bone mineral density) and BV/TV (bone volume to tissue volume) in control and hind-limb unloading mice group. Scale bar, 1 mm; *n* = 6 mice per group. (**b**) Representative images showing new bone formation assessed and dynamic histomorphometric analysis of MAR (mineral apposition rate) by double calcein labeling in trabecular bone (left) and cortical bone (right) of distal femur from control and hind-limb unloading mice group. Upper scale bar, 100 μm; lower scale bar, 25 μm; *n* = 6 mice per group. (**c**) Real-time PCR analysis of *Alp*, *ColIα1*, and *Fndc5* expressions in tibias of control and hind-limb unloading mice for 28 days; *n* = 4 mice per group. (**d**) Representative images of alkaline phosphatase (ALP) staining of primary osteoblasts cultured in random position machine for 48 h. Scale bar, 5 mm. (**e**) Real-time PCR analysis of osteogenic gene *Alp*, *ColIα1*, and *Fndc5* expressions in primary osteoblasts after random position machine (RPM) for 48 h; *n* = 3. *Gapdh* was used as the internal control for messenger RNA (mRNA). Ctr: control, SM: simulated microgravity. All data are the mean ± SD. Statistical differences between the two groups were determined by the Student’s *t*-test; * *p* < 0.05, ** *p* < 0.01.

In this study, the hind-limb unloading mice model was used to simulate microgravity effects on bone in vivo. Micro-computed tomograph (MicroCT) was used to analyze the structure and parameters (BMD, BV/TV) of distal femurs trabecular in hind-limb unloading mice and control mice. The results showed that simulated microgravity (SM) led to bone loss in mice femurs (Figure 1a). Moreover, the mineral apposition rates (MAR, bone formation-related parameter) of trabecular bone and cortical bone in hind-limb unloading mice femur were lower than those in the control group (Figure 1b). Importantly, not only were expressions of osteogenic genes such as alkaline phosphatase (*Alp*) and collagen type I alpha 1 (*ColIα1*) respectively downregulated by 93.9% and 62.4%, but the expression of *Fndc5* (the irisin precursor) was also significantly decreased in tibias of hind-limb unloading mice compared to control mice in vivo (Figure 1c).

In vitro, we investigated the inhibitory effects of simulated microgravity (SM) on osteoblast differentiation using a random position machine (RPM) which rotated biological samples along two independent axes to change their orientation in random modes [6]. The images of ALP staining showed that the ALP activity of primary osteoblasts was inhibited under simulated microgravity condition (Figure 1d). Moreover, the expressions of *Alp*, *ColIα1*, and *Fndc5* were also decreased by 68.7%, 68.9%, and 48.8% in primary osteoblasts after RPM simulated microgravity treatment, respectively (Figure 1e). The preceding results suggest that irisin was insufficient under simulated microgravity and positively correlated with bone formation and osteoblast differentiation.

### 2.2. r-Irisin Promotes Primary Osteoblast Differentiation

**Figure 2:**
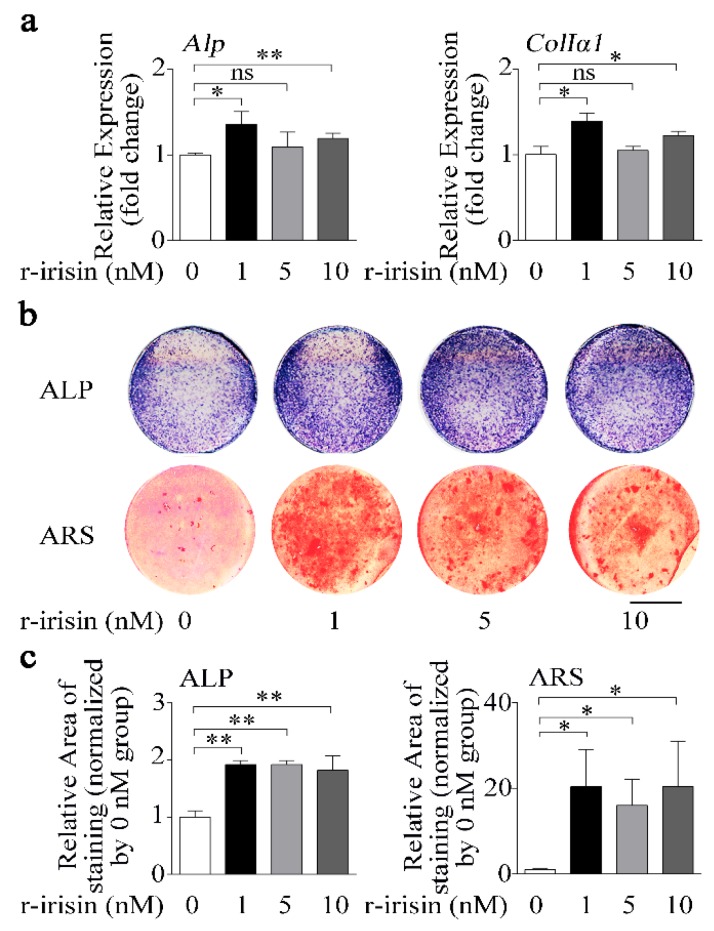
Lower dose of r-irisin promotes primary osteoblast differentiation. (**a**) Real-time PCR analysis of *Alp* and *ColIα1* in primary osteoblasts after treatment with either different dose of r-irisin or PBS for 48 h; *n* = 3. (**b**) Representative images of ALP staining (upper) and Alizarin red staining (ARS) (lower) in primary osteoblasts after treatment with either different dose of r-irisin or PBS for 48 h and 14 d, respectively. Scale bar, 5 mm. (**c**) Quantification of ALP (left) and ARS (right) staining areas in primary osteoblasts after treatment with treatment with either different dose of r-irisin or PBS for 48 h and 14 d, respectively; *n* = 3. *Gapdh* was used as the internal control for mRNA. All data are the mean ± SD. Statistical differences between the two groups were determined by the Student’s *t-*test; * *p* < 0.05, ** *p* < 0.01, ns, no significant difference.

To examine the function of irisin in regulating osteoblast differentiation, we treated primary osteoblasts with either different doses of recombinant irisin (r-irisin) or phosphate buffer saline (PBS). Real-time PCR results showed that 1 nM and 10 nM r-irisin could effectively increase osteogenic marker gene (*Alp* and *ColIα1*) mRNA levels, while 5 nM r-irisin only slightly increased expressions of *Alp* (9.4%) and *ColIα1* (5.0%). Furthermore, the increase for 1 nM r-irisin was higher than that for 5 nM and 10 nM r-irisin (Figure 2a). In addition, ALP activity and mineralization are important marker steps of osteoblast differentiation; thus, we tested ALP activity and mineralized nodule formation of primary osteoblasts after treating with different doses of r-irisin. ALP-positive blue-violet complexes (upper) and Alizarin red-stained mineralized nodules (lower) were obviously increased in r-irisin groups (Figure 2b,c). All these results together indicated that different doses of r-irisin, especially lower doses of r-irisin (1 nM), promoted primary osteoblast differentiation.

### 2.3. Higher Dose of r-Irisin Promotes Primary Osteoblast Proliferation

**Figure 3:**
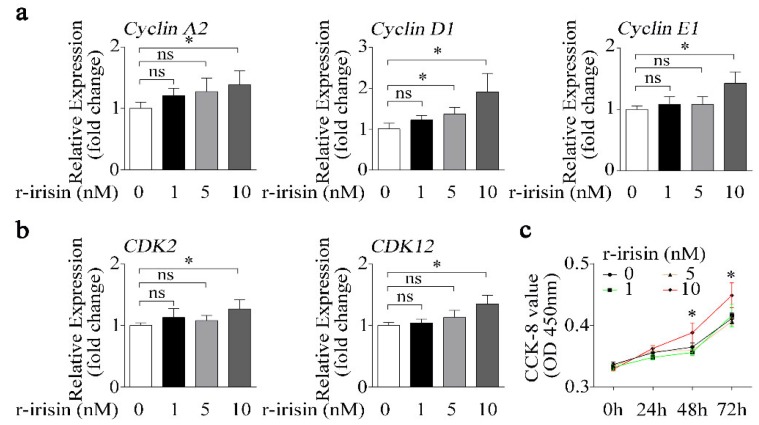
Higher dose of r-irisin promotes primary osteoblast proliferation. (**a**) Real-time PCR analysis of *Cyclin A2*, *Cyclin D1*, and *Cyclin E1* in primary osteoblasts after treatment with a different dose of either r-irisin or PBS for 48 h; *n* = 3. (**b**) Real-time PCR analysis of *CDK2* and *CDK12* in primary osteoblasts after treatment with a different dose of either r-irisin or PBS for 48 h; *n* = 3. (**c**) Cell counting kit (CCK-8) was applied to detect proliferation in primary osteoblasts after treatment with a different dose of either r-irisin or PBS for 0, 24, 48, and 72 h; *n* = 5. *Gapdh* was used as the internal control for mRNA. All data are the mean ± SD. Statistical differences between the two groups were determined by the Student’s *t-*test; * *p* < 0.05, ns, no significant difference.

Not only does osteoblast differentiation indicate osteogenic activity, but osteoblast proliferation is also another important indicator of osteogenic effect. In order to demonstrate the exclusive role of r-irisin in osteoblast differentiation, the effect of r-irisin on osteoblast proliferation should be excluded. Therefore, we treated primary osteoblasts with different doses of either r-irisin or PBS, and found that the expressions of cell proliferation-related genes including cyclin families (*CyclinA2*, *CyclinD1*, *and CyclinE1*), and cyclin-dependent kinase families (*CDK2* and *CDK12*) were gradually upregulated with the increase in r-irisin concentration (Figure 3a,b). Furthermore, the Cell Counting Kit-8 (CCK-8) assay was performed to test the effect of different doses of r-irisin on primary osteoblast proliferation. The value of optical density (OD) 450 nm in primary osteoblasts treated with 10 nM r-irisin was obviously higher than other treatment groups at 48 h and 72 h (Figure 3c). From the above results (Figure 2 and Figure 3), we found that a higher dose of r-irisin (10 nM) promotes osteoblast differentiation and proliferation, but a lower dose of r-irisin (1 nM) only promotes osteoblast differentiation, with no significant effects on osteoblast proliferation. Therefore, we chose a lower dose of r-irisin (1 nM) to further investigate the gain of function of irisin on osteoblast differentiation under simulated microgravity condition.

### 2.4. r-Irisin Partly Prevents the Decrease of Osteoblast Differentiation Induced by Simulated Microgravity

**Figure 4:**
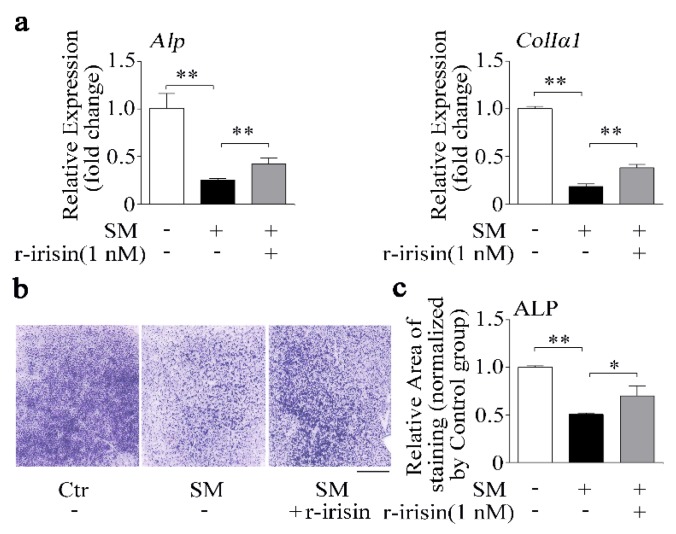
r-Irisin in part prevents the decrease of osteoblast differentiation induced by simulated microgravity. (**a**) Real-time PCR analysis of *Alp* and *ColIα1* in primary osteoblasts after treatment with either 1 nM r-irisin or PBS for 48 h under simulated microgravity; *n* = 3. (**b**) Representative images of ALP staining of primary osteoblasts after treatment with either 1 nM r-irisin or PBS for 48 h under simulated microgravity. Scale bar, 5 mm. (**c**) Quantification of staining areas in primary osteoblasts after treatment with either 1 nM r-irisin or PBS for 48 h under simulated microgravity; *n* = 3. *Gapdh* was used as the internal control for mRNA. Ctr: control; SM: simulated microgravity. All data are the mean ± SD. Statistical differences between the two groups were determined by the Student’s *t-*test; * *p* < 0.05, ** *p* < 0.01.

Due to irisin level being decreased under simulated microgravity condition (Figure 1), we added r-irisin (1 nM) into primary osteoblasts prior to subjecting the cells to RPM-simulated microgravity for 48 h. Osteogenic marker gene mRNA expressions and ALP activity were tested. After 48 h, r-irisin rescued the decrease of *Alp* and *ColIα1* mRNA expressions induced by simulated microgravity (*p* < 0.01, Figure 4a). In addition, the ALP staining results confirmed that reduction of ALP activity caused by simulated microgravity was also counteracted by r-irisin (1 nM) in primary osteoblasts (Figure 4b,c). The results indicated that r-irisin could partly prevent the decrease of primary osteoblast differentiation induced by simulated microgravity.

### 2.5. r-Irisin Promotes β-Catenin Expression to Regulate Osteoblast Differentiation under Simulated Microgravity Condition

**Figure 5:**
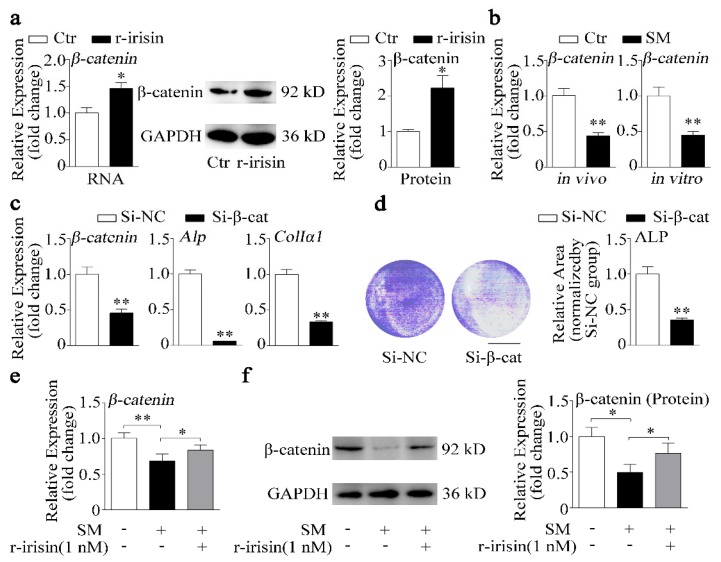
r-Irisin promotes β-catenin expression to regulate osteoblast differentiation under simulated microgravity condition. (**a**) Real-time PCR and Western blot analysis of β-catenin and quantification of β-catenin protein level in primary osteoblasts after treatment with either 1 nM r-irisin or PBS for 48 h; *n* = 3. (**b**) Real-time PCR analysis of *β-catenin* expression in tibias of control and hind-limb unloading mice for 28 days in vivo (*n* = 4) or in primary osteoblasts after random position machine for 48 h in vitro (*n* = 3). (**c**) Real-time PCR analysis of *β-catenin*, *Alp*, and *ColIα1* expressions in MC3T3-E1 cells after treatment with either β-catenin small interfering RNA (siRNA) (Si-β-cat) or siRNA negative control (NC) (Si-NC) for 48 h; *n* = 3. (**d**) Representative images of ALP staining and quantification of staining areas in MC3T3-E1 cells after treatment with either Si-β-cat or Si-NC for 48 h. Scale bar, 5 mm; *n* = 3. (**e**) Real-time PCR analysis of *β-catenin* in primary osteoblasts after treatment with either 1 nM r-irisin or PBS for 48 h under simulated microgravity; *n* = 3. (**f**) Western blot analysis and quantification of β-catenin protein level in primary osteoblasts after treatment with either 1 nM r-irisin or PBS for 48 h under simulated microgravity; *n* = 3. *Gapdh* was used as the internal control for mRNA. Ctr: control; SM: simulated microgravity; Si-β-cat: β-catenin siRNA; Si-NC: siRNA NC. All data are the mean ± SD. Statistical differences between the two groups were determined by the Student’s *t*-test; * *p* < 0.05, ** *p* < 0.01.

Irisin was reported to exert inhibitory effect on adipogenesis through regulating the Wnt/β-catenin signaling pathway [37], which is a key signaling pathway in controlling osteoblast differentiation and bone formation [38]. Colaianni et al. demonstrated that r-irisin could increase β-catenin expression during differentiation of bone marrow stromal cells [27]. Therefore, we hypothesized that irisin might promote osteoblast differentiation through altering β-catenin expression. To investigate the role of irisin in β-catenin signaling, we treated primary osteoblasts with 1 nM r-irisin. As shown in Figure 5a, r-irisin significantly promoted mRNA and protein expressions of β-catenin. Moreover, β-catenin expression was downregulated under simulated microgravity in vivo and in vitro (Figure 5b). To further examine the role of β-catenin in osteoblast differentiation, β-catenin small interfering RNA (siRNA) (Si-β-cat) was used to knockdown β-catenin expression in osteoblast. Expressions of *β-catenin* and osteogenic marker genes (*Alp* and *ColIα1*) were downregulated by more than 50% after treatment with Si-β-cat, compared to treatment with siRNA negative control (Si-NC) (*p* < 0.01, Figure 5c). In addition, ALP staining results revealed that ALP activity was also suppressed by knockdown β-catenin (Figure 5d). r-Irisin promoted β-catenin expression which was decreased under simulated microgravity, and β-catenin also promoted osteoblast differentiation. Thus, we hypothesized that r-irisin might promote osteoblast differentiation through regulating β-catenin expression under simulated microgravity. We found that the decrease of β-catenin mRNA and protein levels induced by simulated microgravity was partly prevented by r-irisin (Figure 5e,f).

### 2.6. β-Catenin Overexpression Recovers Osteoblast Differentiation Reduction Induced by Simulated Microgravity

**Figure 6:**
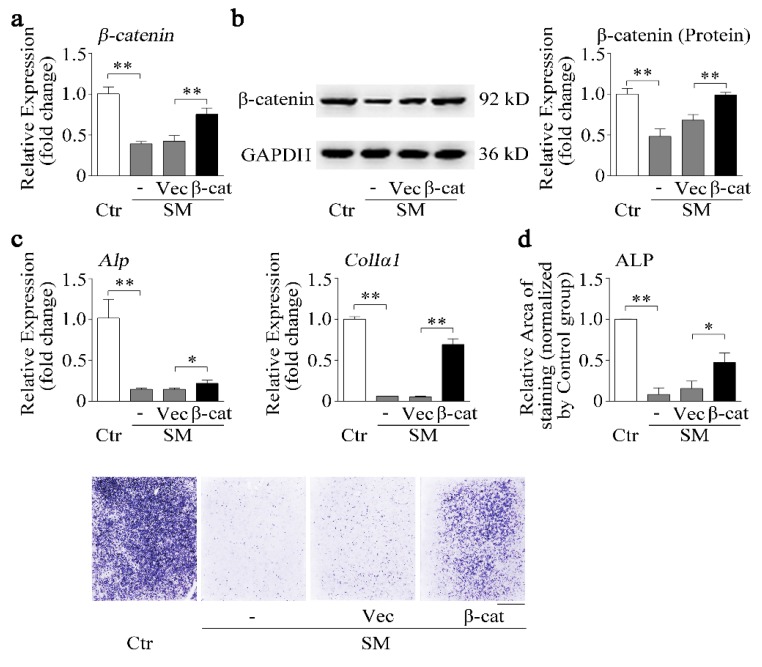
β-catenin overexpression recovers osteoblast differentiation reduction induced by simulated microgravity. (**a**) Real-time PCR analysis of *β-catenin* in MC3T3-E1 cells after treatment with either the pcDNA3.1-β-catenin plasmid (β-cat) or pcDNA3.1 blank vector (Vec) under RPM simulated microgravity for 48 h; *n* = 3. (**b**) Western blot analysis and quantification of β-catenin protein level in MC3T3-E1 cells after treatment with either the β-cat plasmid or blank vector under RPM simulated microgravity for 48 h; *n* = 3. (**c**) Real-time PCR analysis of *Alp* and *ColIα1* in MC3T3-E1 cells after treatment with the β-cat plasmid or blank vector under RPM simulated microgravity for 48 h; *n* = 3. (**d**) Representative images of ALP staining and quantification of staining areas in MC3T3-E1 cells after treatment with the β-cat plasmid or blank vector under RPM simulated microgravity for 48 h. Scale bar, 5 mm. *Gapdh* was used as the internal control for mRNA. Ctr: control; SM: simulated microgravity; β-cat: pcDNA3.1-β-catenin plasmid; Vec: pcDNA3.1 blank vector. All data are the mean ± SD. Statistical differences between the two groups were determined by the Student’s *t-*test; * *p* < 0.05, ** *p* < 0.01.

We know that simulated microgravity inhibited irisin and β-catenin expression. Furthermore, r-irisin could partially counteract the reduction of osteoblast differentiation (Figure 4) and the decrease of β-catenin expression (Figure 5) induced by simulated microgravity. Hence, the effects of β-catenin on osteoblast differentiation under simulated microgravity should be further investigated. Osteoblasts were transfected with the pcDNA3.1-β-catenin plasmid (β-cat) or blank pcDNA3.1 vector (Vec) for 12 h and then exposed to RPM microgravity for 48 h. β-Catenin mRNA and protein levels were partially rescued by the pcDNA3.1-β-catenin plasmid (*p* < 0.01, Figure 6a,b). The osteogenic marker gene (*Alp* and *ColIα1*) mRNA expressions and ALP activity were significantly decreased by simulated microgravity; nevertheless, the decrease was partially attenuated by the pcDNA3.1-β-catenin plasmid under simulated microgravity (*p* < 0.05 or 0.01, Figure 6c,d).

Taken together, r-irisin counteracted osteoblast differentiation reduction induced by simulated microgravity in part through increasing β-catenin expression.

## 3. Discussion

Disuse osteoporosis is a common bone disease, which can be prevented by physical activity (exercise) [39]. Irisin, a novel exercise-induced myokine, was shown to stimulate osteoblast differentiation and bone formation [23,24,25,26,27]. Notably, the novel and most significant finding of this study is that we identified that irisin was downregulated under simulated microgravity and negatively correlated with bone formation and osteoblast differentiation in vivo and in vitro. Furthermore, r-irisin partly counteracted osteoblast differentiation reduction through increasing β-catenin expression under simulated microgravity. Our finding may reveal a novel mechanism for musculoskeletal crosstalk, and it provides a new therapeutic strategy for the pathological osteoporosis and muscle atrophy induced by microgravity.

Microgravity during spaceflight causes disruption of intracorporal bone homeostasis. Notably, there are precipitous declines (more than 10%) in areal bone mineral density detected in the hip and spine in some long-duration astronauts that typically fly 180-day missions in space [40]. The hind-limb unloading mice model is widely used to simulate microgravity effects on the musculoskeletal system in vivo. Recent evidence suggests that musculoskeletal muscle talks to bone via a finely tuned network of molecules termed myokines, such as irisin [20]. In this study, we found that bone formation-related parameters (BMD, BV/TV, MAR) and osteogenic marker genes (*Alp* and *ColIα1*) were inhibited in hind-limb unloading mice. Furthermore, the expression of Fndc5 (irisin precursor) was also significantly downregulated in tibias of hind-limb unloading mice (Figure 1a–c). Kawao et al. demonstrated that Fndc5 mRNA level was decreased in the soleus muscle of hind-limb unloading mice, and regression analysis revealed that the Fndc5 level of soleus muscle was positively related to tibia trabecular BMD [28]. The decrease of osteoblast differentiation is a key reason for bone loss induced by simulated microgravity. Fndc5 level was also decreased in primary osteoblasts under RPM simulated microgravity in vitro (Figure 1d,e). It is suggested that irisin may be as a musculoskeletal crosstalk regulator of bone formation and osteoblast differentiation under simulated microgravity.

Irisin, as an identified peptide hormone, is the cleavage product of Fndc5, released from myocytes into the bloodstream after physical exercise [20]. Irisin was reported to act as a link between muscles and other tissues or organs, especially for bone as a muscle neighboring tissue [20,23]. Colaianni’s study demonstrated that a low cumulative weekly dose of r-irisin (100 μg/kg) was beneficial to young male mice cortical bone mass and bone bending strength [27]. Moreover, BMD of both the cortical and the trabecular bone volume fraction (BV/TV) was also prevented by r-irisin injection in hind-limb unloading mice [26]. Recent studies verified that irisin served as a positive regulator in osteoblast differentiation or proliferation [24,25]. In our study, we found that the osteogenic marker genes (*Alp* and *ColIα1*) and proliferation-related genes (*CyclinA2*, *CyclinD1, CyclinE1*, *CDK2*, and *CDK12*), ALP activity, and calcium deposition were increased after treatment with r-irisin in primary osteoblasts (Figure 2 and Figure 3). Increasing evidence demonstrates that r-irisin effectively promoted the expressions of osteoblastic transcription regulators and osteoblast differentiation markers, ALP activity, and calcium deposition and proliferation in different osteogenic cells including calvaria primary osteoblasts [25], MC3T3-E1 cells [25,41,42], and bone marrow stromal cells (BMSCs) [24,26,27]. Our results indicated that r-irisin promoted primary osteoblast differentiation and proliferation, which is consistent with the above-reported studies. Under simulated microgravity condition, we found that the decrease of primary osteoblast differentiation was rescued by r-irisin in vitro (Figure 4). Furthermore, Colaianni et al. found that r-irisin not only prevented bone loss induced by hind-limb unloading in vivo, but also restored osteoblastogenesis in ex vivo cultures from unloaded mice [26]. These results suggest that r-irisin plays critical roles in osteoblast differentiation and bone formation under simulated microgravity environment.

To investigate the mechanism underlying how r-irisin increased osteoblast differentiation, we analyzed signaling pathways which irisin regulated based on reported studies, and found that irisin could modulate Notch [43], focal adhesion kinase (FAK) – extracellular signal-regulated kinase (Erk) – mitogen-activated protein (MAP) [23,25,44,45], reactive oxygen species (ROS) [46,47], phosphatidylinositol-3-kinase (PI3K) – v-akt murine thymoma viral oncogene homolog 1(AKT) [48], and Wnt/β-catenin [27,37,45] signaling pathways. Significantly, it is reported that irisin inhibited adipogenesis through regulating the Wnt/β-catenin signaling pathway [37]. In addition, Wnt antagonists sclerostin and dickkopf-1 were downregulated in MLO-Y4 cells after treatment with r-irisin [45]. Interestingly, our data demonstrated that r-irisin promoted β-catenin mRNA and protein expression (Figure 5a). Colaianni et al. showed that r-irisin increased β-catenin expression during BMSC osteogenic differentiation [27]. Furthermore, the decrease of β-catenin expression caused by simulated microgravity was prevented by r-irsin in osteoblasts (Figure 5). This evidence strongly suggests that r-irisin promoted osteoblast differentiation in part depending on β-catenin.

Recently, canonical biochemical components such as the Wnt signaling pathway and β-catenin were reported to present mechanical sensitivity [49]. Previous studies showed that Wnt/β-catenin signaling is a key signaling pathway in osteoblast differentiation and bone formation [33,50,51]. We also found that knockdown of β-catenin inhibited osteoblast differentiation in MC3T3-E1 cells (Figure 5c,d). In osteoblasts, mechanical stimulation causes a rapid, transient accumulation of active β-catenin in the cytoplasm and its translocation into the nucleus [34]. Furthermore, we reported that β-catenin reduction caused by simulated microgravity (Figure 5b) [14] and overexpression of β-catenin partially attenuated the negative effects of simulated microgravity on osteoblast differentiation (Figure 6). Thus, we wonder whether β-catenin acts as a mechano-mediator in the processing of r-irisin, by regulating osteoblast differentiation under simulated microgravity.

Overall, we demonstrated the possible mechanism of r-irisin, which promoted osteoblast differentiation under simulated microgravity. In detail, the irisin precursor was decreased under simulated microgravity, and r-irisin promoted osteoblast differentiation by increasing β-catenin expression. Moreover, r-irisin or overexpression of β-catenin partially attenuated the negative effects of simulated microgravity on osteoblast differentiation in vitro. Our findings indicate that the mechanism of r-irisin involves controlling osteoblast differentiation, which may provide a novel therapy for the prevention of osteoporosis and other musculoskeletal disorders by microgravity. Furthermore, irisin can be stimulated by physical activity for the prevention and even future treatment of osteoporosis in genetic conditioning, which still requires further research. Moreover, it should be noted that irisin could promote osteoclast precursor cell proliferation but inhibit osteoclast differentiation [52], which was also altered by microgravity. The muscle–osteoblast–osteoclast crosstalk under microgravity might depend on irisin, which needs further elucidation.

## 4. Materials and Methods

### 4.1. Hind-Limb Unloading Mice Model (Simulated Microgravity In Vivo)

Two-month-old male Balb/c mice were purchased from the Laboratory Animal Center of the Fourth Military Medical University (Xi’an, Shanxi, China). The mice were kept for four weeks with hind limbs suspended. The hind limb suspension procedure was achieved by tail suspension as previously described [53]. In brief, mice were kept in standard cages with maintained suspension position at about a 30° angle. This maneuver permitted the animals to have ad libitum access to food and water. The animal’s overall appearance, drinking and eating habits, and tail were checked two times per day. All mice were injected intraperitoneally with green-fluorescent calcein (Sigma, St. Louis, MO, USA; 10 mg/kg body weight) in a time sequence of 10 d and 2 d before euthanasia. After euthanasia, experiments were conducted by harvesting bilateral femurs, which were processed for micro-computed tomography (microCT) and real-time PCR analysis (*n* = 6 mice per group). All the experimental procedures used in the present study were approved by the Animal Care and Experimental Safety of Northwestern Polytechnical University.

### 4.2. Micro-Computed Tomography (microCT) Analysis

Distal femurs were scanned with an eXplore Locus SP microCT device (General Electric, Milwaukee, WI, USA) at an isotropic resolution of 8 μm. Briefly, mice were anesthetized and sacrificed to isolate femurs without muscle and connective tissue, and cleaned femurs were fixed in 4% paraformaldehyde (PFA) and stored at 4 °C. The scanning protocol was set as follows: X-ray source energy 80 kVp/80 μA, angle of increment 0.5°, exposure time 3000 ms/frame, scanning time 120 min. Volumes of interest (VOIs) were selected for trabecular bone for each sample after three-dimensional (3D) reconstruction, in which trabecular VOIs were selected 0.5 mm above distal growth plate (indicated by coronal plane) at an axial height of 1.0 mm, and the Advanced Bone Analysis Application (General Electric, Milwaukee, WI, USA) was used to calculate stereological parameters including bone mineral density (BMD) and bone volume to tissue volume (BV/TV) for trabecular microarchitecture. Detailed procedures and operations were performed according to published literature [54].

### 4.3. RNA Extraction and Real-Time PCR

Total RNA was used as a template for double-stranded complementary DNA (cDNA) synthesis (PrimeScript™ RT reagent Kit, Takara, Dalian, China). SYBR^®^ Premix Ex Taq™ II (Takara, Dalian, China) was applied for the quantitative RT-PCR. Total RNA was extracted from individual samples of femurs or cells by means of TRIzol reagent (Invitrogen, Carlsbad, CA, USA). For mRNA, cDNA was reverse-transcribed using the PrimeScript RT reagent kit (TaKaRa, Dalian, China). Gene expression was then examined through real-time PCR with the SYBR Premix Ex Taq II kit (TaKaRa, Dalian, China), and real-time PCR analysis was performed using the Thermal Cycler C-1000 Touch system (BIO-RAD CFX Manager, Hercules, CA, USA). All amplifications were normalized to *Gapdh*. Data were analyzed using the comparative Ct method (2^−ΔΔCt^) and expressed as fold change compared to corresponding control. All primer sequences are listed in Table 1.

### 4.4. Preparation of Primary Osteoblasts Isolated from Long Bone

We prepared and cultured mouse primary osteoblasts according to previously reported protocols (*Bone research protocol p22-23*). Primary osteoblasts were isolated from the femurs of two-month-old male mice. Briefly, femurs without muscle and connective tissue had their bone marrow thoroughly flushed out with PBS, using a 5-mL syringe and a 27-gauge needle, before cutting the clean diaphyses into little pieces of approximately 1–2 mm using scissors. The bone pieces were washed several times with PBS, and incubated three times in 0.1% collagenase (Life Technologies, Carlsbad, CA, USA) and 0.25% trypsin (Life Technologies, Carlsbad, CA, USA) at 37 °C in a shaking water bath in order to remove all remaining soft tissue and adherent cells, every 30 min. From the fourth time to the seventh time, the cells were collected and cultured with alpha Modified Eagle’s Medium (α-MEM) containing 10% fetal bovine serum (FBS, Corning, Steuben, NY, USA), and 1% penicillin and streptomycin (Amresco, Fried, WA, USA). Medium was changed three times per week, and, after approximately 7–10 days, cells reached subconfluency, at which point they could be used for experiments.

### 4.5. Cell Culture

The primary osteoblasts were cultured in α-MEM (Gibco, Carlsbad, CA, USA) supplemented with 10% FBS (Corning, Steuben, NY, USA), and 1% penicillin and streptomycin (Amresco, Fried, WA, USA). The primary osteoblasts were maintained under standard cell culture conditions of 5% CO_2_ and 95% humidity and were not used beyond six passages. For the experiments, confluent cells were removed using 0.25% trypsin containing 10 mM ethylene diamine tetraacetic acid (EDTA). For the osteoblast differentiation experiment, primary osteoblasts were cultured in 24-well plates at 10^5^ cells per well with osteogenic medium containing 10% FBS (Biological Industries, Migdal Haemek, Israel), 1% penicillin and streptomycin, 50 μg/mL of ascorbic acid, and 10 mM β-glycerophosphate.

The murine preosteoblast cell line MC3T3-E1 was generously provided by Dr. Hong Zhou of the University of Sydney. The MC3T3-E1 cells were cultured in α-MEM (Gibco, Carlsbad, CA, USA) supplemented with 10% FBS (Biological Industries, Migdal Haemek, Israel), and 1% penicillin and streptomycin (Amresco, Fried, WA, USA). The cell cultures were incubated at a humidified, 37 °C, 5% CO_2_ incubator.

### 4.6. Random Positioning Machine (Simulated Microgravity In Vitro)

A desktop random positioning machine (RPM, the Center for Space Science and Applied Research of Chinese Academy of Sciences) was used to simulate microgravity conditions for the cell culture, as described previously [6,7]. The cell culture vessel was fixed on the inner frame, and the RPM was placed inside a 37 °C incubator. For simulated microgravity studies, cells were seeded at a density of 1 × 10^4^ cells/cm^2^ on glass coverslips in a 90-mm dish and incubated at 37 °C. RPM culture flasks filled with growth medium (air bubbles avoided) were tightly capped and mounted in the inner frame of the RPM. The machine was operated in a random mode of speed (0–8 rpm) and direction including both inner and outer frames for 48 h. Cells of a static control group were cultured in the same 37 °C incubator without rotation.

### 4.7. Alkaline Phosphatase Staining

Alkaline phosphatase (ALP) staining was performed with the BCIP/NBT alkaline phosphatase color development kit (Beyotime, Shanghai, China) according to the manufacturer’s instruction [14,33]. Briefly, cells were carefully rinsed with PBS and fixed with neutral buffered formalin (10%) for 15 min. The fixed cells were rinsed with PBS again and then BCIP/NBT liquid substrate was added to each well. Finally, cells were washed with ddH_2_O after the color turning blue/purple. The stained cell cultures were imaged by a scanner.

### 4.8. r-Irisin Treatment

r-Irisin was purchased from Phoenix Pharmaceuticals Inc. (Burlingame, CA, USA), which was dissolved in sterile PBS. For osteoblast differentiation, primary osteoblasts were seeded in plates at a cell density of 2 ×10^4^ cells/cm^2^. The cells were cultured for 12 to 24 h to reach 80%–90% confluency, at which time different doses of r-irisin were added. The osteogenic medium with r-irisin was changed every two days.

For osteoblast proliferation, primary osteoblasts were seeded in 12-well plates at a cell density of 4 × 10^4^ cells per well. The cells were cultured for 12 to 24 h to reach 40%–50% confluency, at which time the different doses of r-irisin were added. The cells were collected for real-time PCR analysis.

### 4.9. Alizarin Red Staining

Cells were fixed with neutral buffered formalin (10%) for 15 min. The fixed cells were rinsed with PBS and stained with 0.5% Alizarin red s (Sigma-Aldrich, St. Louis, MO, USA) solution (pH 4.2) for 15 min at room temperature. After being washed with ddH_2_O on a shaking platform four times, 5 min/time, the mineralized nodules were imaged by a scanner.

### 4.10. Cell Proliferation Assay

Cell proliferation activity was measured using the Cell Counting Kit-8 (CCK-8) (Biosharp, Shanghai, China). The primary osteoblasts were seeded in 96-well plates at a cell density of 2000 cells with 100 μL medium per well. After adherence, the cells were added with different doses of r-irisin or PBS, then cultured for 0, 1, 2, and 3 d with drug. Next, 10 μL of CCK-8 solution was added into each well, followed by incubation with cells for 1 h. The absorbance was recorded at 450 nm. All groups performed at least five replicates.

### 4.11. Western Blot Analysis

Cells were lysed in Cell Lysis Buffer for Western and IP (Beyotime, Shanghai, China) supplemented with 1% Protease inhibitor Cocktail Set III (Calbiochem, Darmstadt, Germany) on ice. Protein fractions were collected by centrifugation at 15,000× *g*, 4 °C, for 5 min and subjected to SDS-PAGE, before being transferred to NC membranes (PALL, Port Washington, NY, USA). The membranes were blocked with 5% skim milk and incubated with specific antibodies overnight at 4 °C. HRP-labeled secondary antibody (CoWin Bioscience, Beijing, China) was added and then visualized using a Chemiluminescence Detection System (Promega, Madison, WI, USA) as recommended by the manufacturer. The following primary antibodies were used: β-catenin Rabbit polyclonal antibody (pAb) (1:1000, Cell Signaling Technology, Danvers, MA, USA) and glyceraldehyde 3-phosphate dehydrogenase (GAPDH) Rabbit pAb (1:1000, Servicebio, Wuhan, China).

### 4.12. Cell Transfection

MC3T3-E1 cells were seeded at cell density of 1 × 10^4^ cells/cm^2^ until cultured to reach 80%–90% confluency. Then, the cells were transfected with 100 nM β-catenin siRNA (si-β-cat) by Lipofectamine 2000 (invitrogen, Carlsbad, CA, USA). After transfection for 6 h, serum-free medium was replaced by growth medium. MC3T3-E1 cells were harvested for real-time PCR analysis and alkaline phosphatase staining after 48 h. β-Catenin siRNA (Ribobio, Guangzhou, China) sequences were 5′–CAAGCCUUAGUAAACAUAAdTdT–3′ (positive-sense strand) and 3′–dTdTGUUCGGAAUCAUUUGUAUU–5′ (antisense strand).

For simulated microgravity studies, MC3T3-E1 cells were seeded at a density of 1 × 10^4^ cells/cm^2^ on glass coverslips in a 90-mm dish and incubated at 37 °C. Cells were cultured until to reach 80%–90% confluency and then transfected with 2 μg/mL pcDNA3.1-β-catenin plasmid by Lipofectamine 2000 (invitrogen, Carlsbad, CA, USA). After transfection for 6 h, serum-free medium was replaced by the growth medium. After 12 h, cells were transferred to RPM culture flasks. The blank pcDNA3.1 plasmid was used as a negative control plasmid.

### 4.13. Statistical Analysis

All numerical data were expressed as the mean ± SD. All experiments were performed with at least three replicates. Statistically significant differences were analyzed using Student’s *t*-test with the statistical software Prism (GraphPad Software Inc, La Jolla, CA, USA). A value of *p* < 0.05 was considered significant in all cases (* *p* < 0.05, ** *p* < 0.01).

## Figures and Tables

**Table 1:** The primer sequences for real-time PCR.

Gene Name	Forword (5′–3′)	Reverse (5′–3′)
*Alp* (NM_007431.1)	GTTGCCAAGCTGGGAAGAACAC	CCCACCCCGCTATTCCAAAC
*ColIα1* (NM_007742.3)	GAAGGCAACAGTCGATTCACC	GACTGTCTTGCCCCAAGTTCC
*Fndc5* (NM_027402.4)	GAGCCCAATAACAACAAGG	GAGGATAATAAGCCCGATG
*Cyclin A2* (NM_009828.3)	AGTACCTGCCTTCACTCATTGCTG	TCTGGTGAAGGTCCACAAGACAAG
*Cyclin D1* (NM_007631.2)	CAGCCCTGTTACCTGATACCT	TCCCAAGCACCTCATACTACC
*Cyclin E1* (NM_007633.2)	GCTTCGGGTCTGAGTTCCAA	GGATGAAAGAGCAGGGGTCC
*CDK2* (NM_016756.4)	TGTGCCTCCCCTGGATGAAG	CATCCTGGAAGAAAGGGTGA
*CDK12* (NM_026952.2)	CTGAATAACAGCGGGCAAAT	AGCTCTGGAGGTCGATACCA
*β-catenin* (NM_007614.3)	ACGCTGCTCATCCCACTAAT	AGTTCCGCGTCATCCTGATA
*Gapdh* (NM_008084.2)	TGCACCACCAACTGCTTAG	GGATGCAGGGATGATGTTC

## Abbreviations

AKT	v-akt murine thymoma viral oncogene homolog 1
ALP/Alp	alkaline phosphatase
ARS	Alizarin red staining
BMSCs	bone marrow stromal cells
BMD	bone mineral density
BMP	bone morphogenetic protein
BV/TV	bone volume to tissue volume (bone volume fraction)
CCK-8	Cell Counting Kit-8
CDK2	cyclin-dependent kinase 2
CDK12	cyclin-dependent kinase 12
Ctr	control
ColIα1	collagen type 1 alpha-1
EDTA	ethylene diamine tetraacetic acid
Erk	extracellular signal-regulated kinase
FAK	focal adhesion kinase
FBS	fetal bovine serum
FNDC5/Fndc5	fibronectin type III domain-containing 5
Gapdh/GAPDH	glyceraldehyde 3-phosphate dehydrogenase
MAP	mitogen-activated protein
MAR	mineral apposition rate
microCT	micro-computed tomography
NC	negative control
OD	optical density
pAb	Polyclonal antibody
PBS	phosphate buffer saline
PFA	paraformaldehyde
PI3K	phosphatidylinositol-3 kinase
r-irisin	recombinant irisin
ROS	reactive oxygen species
RPM	random position machine
SM	simulated microgravity
Si-β-cat	β-catenin siRNA
Si-NC	siRNA NC
Vec	pcDNA3.1 blank vector
Wnt	wingless-type MMTV integration site family
α-MEM	alpha Modified Eagle’s Medium
β-cat	pcDNA3.1-β-catenin plasmid
